# Influence of Calcium Silicate and Hydrophobic Agent Coatings on Thermal, Water Barrier, Mechanical and Biodegradation Properties of Cellulose

**DOI:** 10.3390/nano11061488

**Published:** 2021-06-04

**Authors:** Saravanan Chandrasekaran, Remi Castaing, Alvaro Cruz-Izquierdo, L. Janet Scott

**Affiliations:** 1 Centre for Sustainable Chemical Technologies, Department of Chemistry, University of Bath, Claverton Down, Bath BA2 7AY, UK; alcruz99@gmail.com; 2 Department of Chemistry, School of Engineering, Presidency University, Rajanukunte, Itgalpura, Bangalore 560064, India; 3 Material and Chemical Characterisation Facility (MC2), University of Bath, Claverton Down, Bath BA2 7AY, UK; R.Castaing@bath.ac.uk

**Keywords:** α-cellulose, CaSiO_3_, ionic liquid, coatings, hydrophobic agent coatings, biodegradability

## Abstract

Thin films of cellulose and cellulose–CaSiO_3_ composites were prepared using 1-ethyl-3-methylimidazolium acetate (EMIMAc) as the dissolution medium and the composites were regenerated from an anti-solvent. The surface hydrophilicity of the resultant cellulose composites was lowered by coating them with three different hydrophobizing agents, specifically, trichloro(octadecyl)silane (TOS), ethyl 2-cyanoacrylate (E2CA) and octadecylphosphonic acid (ODPA), using a simple dip-coating technique. The prepared materials were subjected to flame retardancy, water barrier, thermal, mechanical and biodegradation properties analyses. The addition of CaSiO_3_ into the cellulose increased the degradation temperature and flame retardant properties of the cellulose. The water barrier property of cellulose–CaSiO_3_ composites under long term water exposure completely depends on the nature of the hydrophobic agents used for the surface modification process. All of the cellulose composites behaved mechanically as a pure elastic material with a glassy state from room temperature to 250 °C, and from 20% to 70% relative humidity (RH). The presence of the CaSiO_3_ filler had no effect on the elastic modulus, but it seemed to increase after the TOS surface treatment. Biodegradability of the cellulose was evaluated by enzyme treatments and the influence of CaSiO_3_ and hydrophobic agents was also derived.

## 1. Introduction

The development of flexible electronic devices is rapidly increasing worldwide and they have great potential for use in the latest technology in the near future. Compared to non-flexible materials, flexible materials have the advantage of being able to make thin, light-weight and user-friendly electronic devices. Recently, various flexible electronic devices such as photovoltaics, batteries, transistors, sensors, light-emitting diodes and electronic skin have been reported [1,2,3,4,5]. The materials used for making flexible substrates are mostly based on synthetic polymers, flexible glass and metal foils [6,7]. Polymer-based flexible materials are made from polyimide, polyethylene terephthalate, polyethylene naphthalate, polycarbonate, polystyrene and polypropylene. Most of these polymers originate from non-renewable petroleum resources and they are non-biodegradable and very difficult to recycle. Therefore, various research groups are working towards achieving the next generation of flexible materials from renewable resources to replace the non-renewable petroleum-based polymers [5,8,9]. However, there are several practical challenges in designing the next generation of flexible substrate materials with the required properties such as chemical resistivity, suitable surface functionality, flammability, mechanical and thermo-mechanical stability and degradability.

Silk, wool, rubber and cellulose are natural polymers that are used in many applications [10,11,12,13,14,15,16]. Among the various types of natural polymers, cellulose is one of the most attractive materials: cellulose has excellent mechanical properties, is the most abundant biomass material, is available from renewable resources, is biocompatible and biodegradable and its surfaces can be readily chemically functionalised. Recently, cellulose-based flexible materials have been used in various electronic devices, for example, in energy storage [17], transistors [18], photovoltaics [19], display [20], actuators [21] and high-performance devices [22,23]. However, the insolubility associated with the non-processability, flammability and hydrophilicity of cellulose restricts its fields of utilisation. In the available reports, numerous methods have been developed to dissolve the cellulose in various solvent mediums, to impart the additional properties to the cellulose-based composite materials [24,25,26,27,28]. However, most of the above methods use hazardous non-recyclable dissolution media, several side-reactions occur during the dissolution process and they are completed at a high cost. Moreover, biopolymers are processed by injection moulding, extrusion, thermoforming and blow moulding [29,30]. During the above processes materials undergo various physicochemical, thermal changes and lose their original properties. Therefore, several researchers have utilised ionic liquids (ILs) as the amenable and environment-friendly solvent to dissolve the cellulose and they found that ILs are the best dissolution medium [25,31,32,33]. Among the various ILs, 1-ethyl-3-methylimidazolium acetate (EMIMAc) has a less sterically hindered cation group (EMIM) and a shorter alkyl chain, which allows it to more easily enter the cellulose to break down the intermolecular and intramolecular hydrogen bonds and accelerate the cellulose dissolution process [33,34,35]. EMIMAc has other advantages including low toxicity, low corrosiveness, low melting point, lower viscosity and biodegradability [35,36].

The flammability of cellulose can be reduced by incorporating various flame retardants (FR) [37,38,39,40,41]. The most common FR materials are mainly halogenated compounds and are not environment-friendly. Therefore, non-halogenated FR is an attractive alternative material to the halogenated FR [42,43,44]. On the other hand, in the literature, several approaches have been reported that fabricate the hydrophobic agent on cellulose-based materials including fluorocoating using plasma techniques, dyeing technologies, graft-on-graft methods, spray-coating, dip-coating, photothiol and chemical vapour deposition (CVD) methods [45,46,47,48]. However, some of the above techniques have limitations including harsh conditions, requiring multi-step procedures, tedious fabrications and expensive materials. Therefore, developing a fast, convenient and scalable method is important to produce hydrophobic surfaces on cellulose-based materials.

To address the aforementioned issues, we employed EMIMAc ionic liquid (IL) as a recoverable and amenable dissolution medium to dissolve the cellulose, CaSiO_3_ as the filler to reduce the flammability, and three different hydrophobic agents to minimize the cellulose’s hydrophilicity. The prepared composite was subjected to chemical, thermal, mechanical and biodegradation studies and the results are reported here.

## 2. Materials and Methods

### 2.1. Experimental Details

#### 2.1.1. Materials

**Scheme 1:**
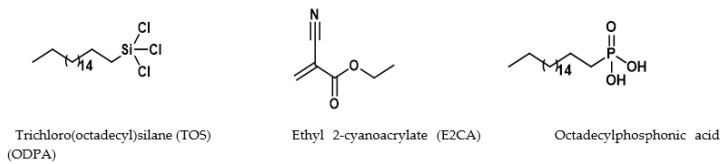
Structure of hydrophobic agents.

α-cellulose (Sigma-Aldrich, Saint Louis, MI, USA) was dried under vacuum (Rotavapor) for 4 h at 80 °C, 1-ethyl-3-methylimidazolium acetate (EMIMAc) (BASF, Ludwigshafen, Germany, Basionics, >95 wt%) was kept under vacuum (Schlenk line) for 4 h at 90 °C. HPLC grade methanol, toluene, tetrahydrofuran (THF), ethyl 2-cyanoacrylate (E2CA), trichloro(octadecyl)silane (TOS) from Sigma-Aldrich, n-octadecylphosphonic acid (ODPA) (97%, Alfa Aesar, Haverhill, MA, USA) and ethanol (VWR Chemicals, Radnor, PA, USA) were used as received. Dried toluene was used to dissolve the trichloro(octadecyl)silane (TOS). See Scheme 1 for the structure of hydrophobic agents. Calcium silicate (CaSiO_3_) (Sigma-Aldrich) was kept under vacuum for 6 h at 110 °C to remove the moisture. Celluclast 1.5 L produced by *Trichoderma reesei* ATCC 26921, citric acid monohydrate, 3,5-dinitrosalicylic acid (DNS), sodium hydroxide, sodium potassium tartrate, phenol and sodium metabisulphite were obtained from Sigma-Aldrich.

#### 2.1.2. Preparation of Cellulose–CaSiO_3_ Composites

In this work, the Brabender plastograph (Duisburg, Germany) was used as the mixer to prepare the cellulose–CaSiO_3_ composite in the presence of EMIMAc as the dissolution medium. The mixer was pre-heated to 90 °C and the blade rotation speed was set to 70 rpm. Cellulose composite with 10 wt% CaSiO_3_ was prepared as follows: 68 g of EMIMAc (85 wt% w.r.t the total weight of α-cellulose and EMIMAc) and 1.3 g of CaSiO_3_ (10 wt% w.r.t the total weight of α-cellulose and CaSiO_3_ used in the dissolution process) were added into the pre-heated mixer and allowed to disperse well in EMIMAc for 5 min. Consequently, 12 g of α-cellulose (15 wt% w.r.t the total weight of α-cellulose and EMIMAc) was divided into 4 × 3 g and added into the mixer consecutively every 5 min. Finally, mixing was allowed for another 10 min at 90 °C. The total mixing time was 30 min. The resultant composite was recovered from the mixer and kept in an air-tight container.

Cellulose-FR composite films were prepared in a hydraulic press equipped with a heating unit (Moore, UK). 14 g of the above-prepared cellulose–CaSiO_3_ composite material was kept between the non-stick bake-o-glide sheets (bake-o-glide is a non-stick baking sheet that is polytetrafluoroethylene (PTFE)-coated fabric) and the pressed film was made by keeping the non-stick baking sheet between the metal plates of the hydraulic press followed by applying pressure (80 psi force at 90 °C). The uniform pressure was maintained for 30 min. In the end, the cellulose film cast on the non-stick baking sheet was removed and subjected to a regeneration process.

The non-stick baking sheet was placed in a methanol bath and left for few minutes. Then the non-stick baking sheet was slowly peeled off in the methanol solvent to obtain the cellulose composite in a continuous film form. The obtained film was placed in another methanol solvent bath to remove EMIMAc completely from the cellulose composites. Finally, the regenerated film was dried at room temperature (RT) using an embroidery hoop as a supporting holder to obtain a non-curly film.

### 2.2. Surface Modification of Cellulose–CaSiO_3_ Composite Film via Hydrophobic Agent Coating

A hydrophobic agent with a weight of 10 wt% was used for the total mass of the composite. Here, a simple dip-coating technique was adopted to coat the composite with the hydrophobic agent.

(i) Cellulose–CaSiO_3_ composite surface coating with TOS

Cellulose composites coated with hydrophobic agent coated were prepared by immersing the cellulose composites in 23 mg (0.003 M) of TOS in 19.8 g of dry toluene solution for 2 or 30 min at room temperature. The whole reaction mixture (except the 2 min samples) was kept under argon atmosphere to protect the TOS solution from moisture. Afterwards, the cellulose composite was removed from the solution and dried at room temperature for 30 min. The same procedure was repeated once again to make sure the surface of the cellulose composites was completely coated with TOS. The resultant surface-modified composites were dried at RT overnight and subjected to further characterisation studies.

(ii) Cellulose–CaSiO_3_ composite surface coating with ODPA and E2CA

ODPA is not completely soluble in THF using a normal stirring method; therefore, ODPA/THF (0.003 M, 20 mg in 19.8 g of THF) solution was sonicated for 20 s and used to coat the cellulose composites. The rest of the procedure is the same as the previous TOS coating procedure.

E2CA (0.003 M, 109 mg in 290.5 g of toluene) is a more reactive monomer and the reaction mixture was prepared in a plastic container as E2CA reacts with glass. The dip-coating was also performed at 4 °C (kept in the fridge). The rest of the procedure is the same as the TOS coating procedure.

### 2.3. Characterization Techniques

Limiting oxygen index (LOI) and flammability tests UL-94 HB (horizontal) were carried out following the standard methods such as ASTM D2863 and ASTM D635-03. The LOI measurements were carried out using Fire Testing Technology, East Grinstead, UK, according to the standard method. The sample was held vertically in the glass chamber of the instrument in which the flow of oxygen and nitrogen mixture gas was controlled. The LOI values of the composites were calculated from the required oxygen concentration in the mixture of gases for burning the composites under the ignition. Samples (composites) for flammability tests were cut into rectangular shape (125 mm × 13 mm) with a thickness of 0.06–0.07 mm. Each cellulose composite film was marked with two lines perpendicular to the longitudinal axis of the bar, 25 ± 1 mm and 100 ± 1 mm from the end that is to be ignited and they were subjected to the horizontal flame test in accordance with the standard method. A sample was clamped horizontally and the burner was used to ignite the samples. The flame retardancy behaviour of the composites was calculated from the time required to burn the defined distance. All the measurements were repeated three times and the results averaged.

Attenuated total reflectance Fourier-transform infrared spectroscopy (ATR-FTIR, Perkin Elmer: Waltham, MA, USA) was recorded in the frequency range of 4000 cm^−1^ to 550 cm^−1^. The surface morphology of uncoated cellulose-FR composites and those coated with the hydrophobic agent were studied using a scanning electron microscope (JEOL JSM-6480, Tokyo, Japan). Contact angle (CA) measurements were conducted using the sessile droplet method in the air at room temperature with 2 µL droplets of de-ionized (DI) water. The cellulose composite was fixed on the glass plate with the support of cello tape and placed on the goniometer and the contact angle was determined 10 s after the water droplet was deposited on the surface of the cellulose composites. Three samples were used and each data point was an average of 10 measurements on each sample. The time-dependent contact angle values were measured in all the hydrophobic agent-coated samples at 10 s, 5 min and 15 min after water droplets were deposited on the surface of the cellulose composites.

A Setsys Evolution TGA 16/18 from Setaram (Caluire, France) was used for the thermogravimetric analysis (TGA); the Calisto program was employed to collect and process the data. Cellulose films were loaded into an alumina crucible and TGA was performed in the temperature range of 30 °C to 400 °C under argon atmosphere at a heating rate of at 5 °C/min. During the heating ramp, evolving gas was transferred from the analytical chamber to a mass spectrometer through a stainless-steel capillary. The mass spectrometer was an Omnistar GSD 320 by Pfeiffer Vacuum (Aßlar, Germany), equipped with a quadrupole mass analyser and a Faraday detector.

A dynamic mechanical analyser DMA1 from Mettler Toledo (Greifensee, Switzerland) was employed for the evaluation of all mechanical properties. The STARe program was used to acquire and process the data. Rectangular sample films with dimensions of 25 mm × 4 mm × 0.06–0.07 mm were clamped in the tension clamping assembly leaving a free length of 10 mm. The cross-section area was then 0.24–0.28 mm^2^. For humidity measurements only, sample films were 10-mm wide, giving a cross-section area of 0.60–0.70 mm^2^. Strain-stress curves were measured in static mode from 0 N to 10 N at a constant load rate of 0.5 N/min and at 25 °C. Creep tests were performed in static mode at 25 °C by applying alternative loads of 1 N and 0 N over a period of 30 s. The elastic moduli and damping factor were measured in dynamic mode at a strain amplitude of 0.1% at a frequency of 1 Hz. For temperature scan measurements, the testing temperature ranged from 25 °C to 250 °C at a heating rate of 2 °C/min. For humidity measurement, the testing temperature was fixed to 30 °C using circulated water, and the humidity was set in the analysis chamber using a modular humidity generator proUmid MHG32 (Ulm, Germany).

### 2.4. Biodegradability Studies

The activity of Celluclast 1.5 L was determined by measuring the soluble reducing sugars using DNS assay [49]. Typical enzymatic hydrolysis consisted of 3 mg of sample, 0.2 mL of sodium citrate buffer (50 mM, pH 5.0) and Celluclast 1.5 L (1 Filter Paper Unit/g of sample) kept for 1 h at 50 °C. All reactions were run in triplicate.

## 3. Results and Discussion

### 3.1. Preparation of Cellulose–CaSiO_3_ Composites

The cellulose–CaSiO_3_ composite was made using EMIMAc as a dissolution medium in a Brabender plastograph mixer, and the film was formed by hydraulic pressing. Finally, the film was regenerated using methanol as an anti-solvent. After the process, EMIMAc and methanol were recovered using the solvent evaporation process. This is a simple method of preparation that uses a relatively low thermal process and is economically affordable, environment-friendly, industrially scalable and an overall quicker process. Further characterisation studies have been carried out on the prepared cellulose–CaSiO_3_ composites.

### 3.2. Characterization of Cellulose–CaSiO_3_ Composites

#### Effect of CaSiO_3_ on Flame Retardant Properties of Cellulose–CaSiO_3_ Composites

A flame retardancy test was conducted on the prepared cellulose–CaSiO_3_ composite. The flame retardancy properties of cellulose without and with 10 wt% CaSiO_3_ were evaluated in terms of LOI and UL-94 HB rating. The results are summarised in Table 1. From LOI data, cellulose without filler is a flammable material and its LOI value is only 19. The LOI value increased from 19 to 20.7 when CaSiO_3_ was added as a flame-retardant. The UL-94 HB test results of the prepared cellulose–CaSiO_3_ composites are also given in Table 1. As can be seen, incorporating CaSiO_3_ considerably reduces the flammability of cellulose. However, CaSiO_3_ is not an efficient filler for cellulose to meet the required FR standard UL-94 HB test.

### 3.3. Surface Coatings of Cellulose–CaSiO_3_ Composites

#### 3.3.1. Preparation of Coated Cellulose–CaSiO_3_ Composites

A hydrophobic cellulose composite was formed by coating the cellulose with a relatively low concentration of a hydrophobic agent (0.003 M). In this work, we have chosen TOS, E2CA and ODPA as the hydrophobic agents to introduce hydrophobicity into the cellulose composites. To ensure the adequate coverage of the hydrophobic agents on the composites, dip-coating was performed two times. The amount of hydrophobic agent in the cellulose composites was calculated by measuring the weight difference between the samples before and after coating with the hydrophobic agents and the calculated amount was in the range of 2–5 wt% irrespective of the nature of the hydrophobic agents. The resultant hydrophobic agent-coated cellulose composites were subjected to further characterisation studies.

#### 3.3.2. ATR-FTIR of Uncoated and Coated Cellulose–CaSiO_3_ Composites

**Figure 1:**
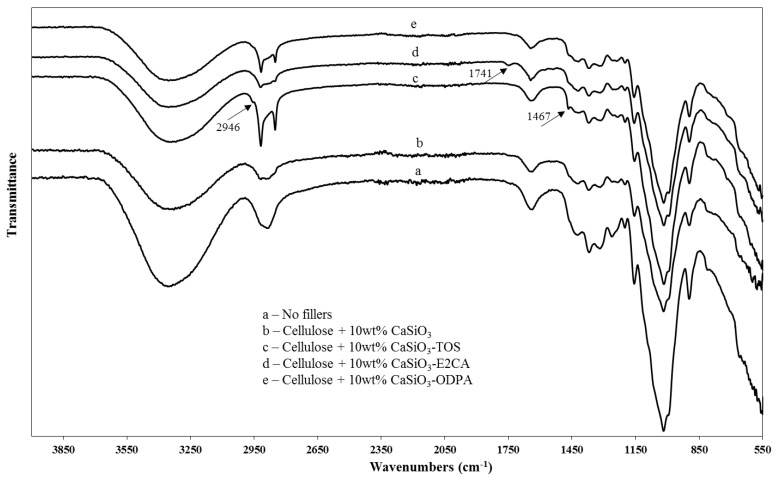
ATR-FTIR of (**a**) cellulose with no fillers, (**b**) cellulose-10 wt% CaSiO_3_ composite, (**c**) cellulose-10 wt%CaSiO_3_ composite–TOS coated, (**d**) cellulose-10 wt% CaSiO_3_ composite–E2CA coated and (**e**) cellulose-10 wt% CaSiO_3_ composite–ODPA coated.

The ATR-FTIR spectra of uncoated and coated cellulose with 10 wt% CaSiO_3_ are shown in Figure 1. The bands at 3353 cm^−1^ and in the range 2927–2879 cm^−1^ are the hydroxyl group and –CH/–CH_2_ stretching vibration of the cellulose, respectively. The band at 1640 cm^−1^ is attributed to the presence of moisture in cellulose. All CaSiO_3_ bands are merged with cellulose and no difference was observed in the cellulose–CaSiO_3_ composites (Figure 1a,b). The TOS-coated cellulose–CaSiO_3_ composite (Figure 1c) has IR bands at 2946, 2918, 2851, 1467 cm^−1^ assigned to the –CH stretching region (–CH_3_ (sym), –CH_2_ (asym), –CH_2_ (sym) and –CH_2_ (bending)) of the octadecyl group from TOS attached with cellulose–CaSiO_3_ composite. The ODPA-coated cellulose–CaSiO_3_ composite (Figure 1d) has similar IR bands as the TOS spectrum. The E2CA-coated cellulose–CaSiO_3_ composite (Figure 1e) has weak IR bands at 2918 and 2854 cm^−1^ (−CH_2_ (asym) and –CH_2_ (sym)) along with an additional band at 1741 cm^−1^ assigned to the –C=O stretching in the ester group of the polymerised form of E2CA (PECA) [50,51].

#### 3.3.3. Water Resistance Characteristics of Cellulose–CaSiO_3_ Composites

**Figure 2:**
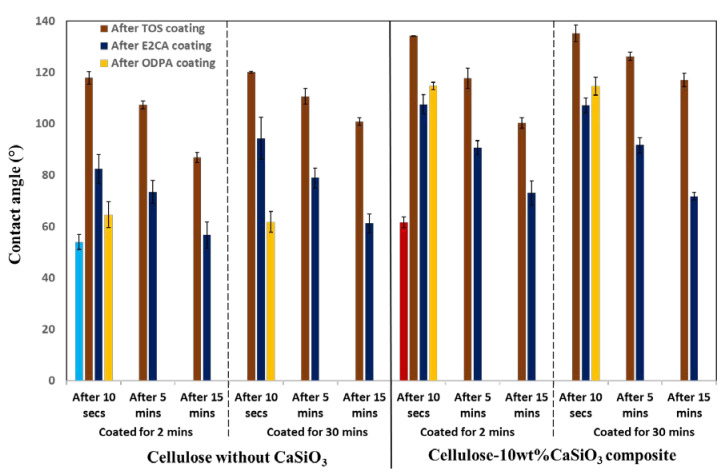
Surface contact angle measurements on cellulose–CaSiO_3_ composites coated with hydrophobising agents TOS, E2CA and ODPA without CaSiO_3_ before coating, with 10 wt% CaSiO_3_.

The water resistance properties of uncoated and coated cellulose composites were evaluated by water contact angle measurements (Figure 2). The as-prepared uncoated cellulose composite with 10 wt% CaSiO_3_ showed a slightly higher contact angle value (62°) than the cellulose without CaSiO_3_ (CA = 54°, Figure 2). This is possibly due to the surface roughness of cellulose being slightly increased in the presence of CaSiO_3_. However, the contact angle value is within the hydrophilic range (CA = 0–90°). Therefore, materials were coated with various types of hydrophobizing agents such as TOS, E2CA and ODPA (Scheme 1).

TOS and ODPA have a long alkyl chain (C18) as a tail, and Si and P with reactive functional groups as a head, respectively. Functional groups in the head of the hydrophobic agents interact with the hydroxyl (OH) groups of cellulose to make a bond while long alkyl chains are oriented towards the upper directions and hinder the access of the water to the cellulose surface. On the other hand, E2CA is a highly reactive monomer that undergoes anionic polymerisation with the hydroxyl (OH) group of cellulose when exposed to moisture present in the atmosphere and makes strong bonds with cellulose [52,53]. The resultant polyethyl 2-cyanoacrylate (PECA) acts as a barrier to moisture and increases the hydrophobicity of the cellulose [54]. Ultimately, the moisture absorption property of cellulose can be controlled by introducing various functional groups into the OH groups of cellulose. In other words, hydrophilicity can be reduced by introducing surface roughness and lowering the surface energy of cellulose by modifying the surface functionality.

Figure 2 shows the variation of contact angles and water resistance properties of cellulose and cellulose with 10 wt% CaSiO_3_ composites depending on the hydrophobic agents. The contact angle value increased from 54° to 118° with TOS as the hydrophobic agent (cellulose without CaSiO_3_ sample coated with TOS for 2 min). The contact angle value was reduced from 118° to 107° (9% loss from the initial CA value) and 87° (26% loss compared to the initial CA value) by extending the time of the water droplet on the surface of the materials (water evaporation was not taken into account) from 10 s to 5 min and 15 min, respectively. This could be due to the hydrolysis of the hydrophobic agent or to the hydrophobic agent’s anchoring nature on the surface of the cellulose not being strong enough to withstand the presence of water molecules, leading to a loss of surface functionality followed by the absorption or penetration of water into the cellulose film. When the hydrophobic agent coating time was extended from 2 min to 30 min, there was no appreciable increase in the contact angle value (contact angle value of 120° for the cellulose sample coated with TOS). However, as shown in Figure 2 the water resistance property of the cellulose increased and CA value reached to111° (7.5% from the initial CA value) and 101° (16% loss from the initial CA value) after 5 min and 15 min, respectively. This may be due to the increase in the interaction between TOS and cellulose while the surface treatment time was extended from 2 min to 30 min and the amount of TOS in the cellulose increased. Contact angle values did not improve appreciably when E2CA was employed as the hydrophobic agent (82°). When the coating time was extended from 2 min to 30 min, the contact angle value further increased to 94°. The water resistance property of the E2CA-coated samples exhibited a similar trend to that of the TOS-coated samples. Noticeably, the contact angle value is close to the value (64°) of the uncoated cellulose when ODPA was used as the hydrophobic agent. This is could be due to the less reactivity and poor addition of ODPA with cellulose.

On the other hand, as shown in Figure 2, the highest contact angle values 134° (TOS), 107° (E2CA) and 115° (ODPA) were observed for cellulose–CaSiO_3_ composites_._ This indicates that CaSiO_3_ may absorb more hydrophobic agents and make strong bonds during the coating process, which explains the higher contact angle value than in the absence of CaSiO_3_ [55,56]. Besides, there was no appreciable improvement in the contact angle value while extending the hydrophobic agent coating time from 2 min to 30 min. The water resistance properties of TOS- and E2CA-coated cellulose–CaSiO_3_ composites exhibited a similar trend as that observed in the absence of CaSiO_3_. On the contrary, we were unable to analyse the water resistance property of the ODPA-coated samples. As mentioned earlier, this may be due to ODPA having less reactivity and poor coordination with the cellulose–CaSiO_3_ composite in the presence of water.

The conductive ink printing studies have been carried out on uncoated and coated cellulose-laponite composite and reported in the literature [54]. Therefore, we did not repeat the same experiment with the prepared cellulose–CaSiO_3_ composite.

#### 3.3.4. Surface Morphology Analysis of Uncoated and Coated Cellulose–CaSiO_3_ Composites by SEM

**Figure 3:**
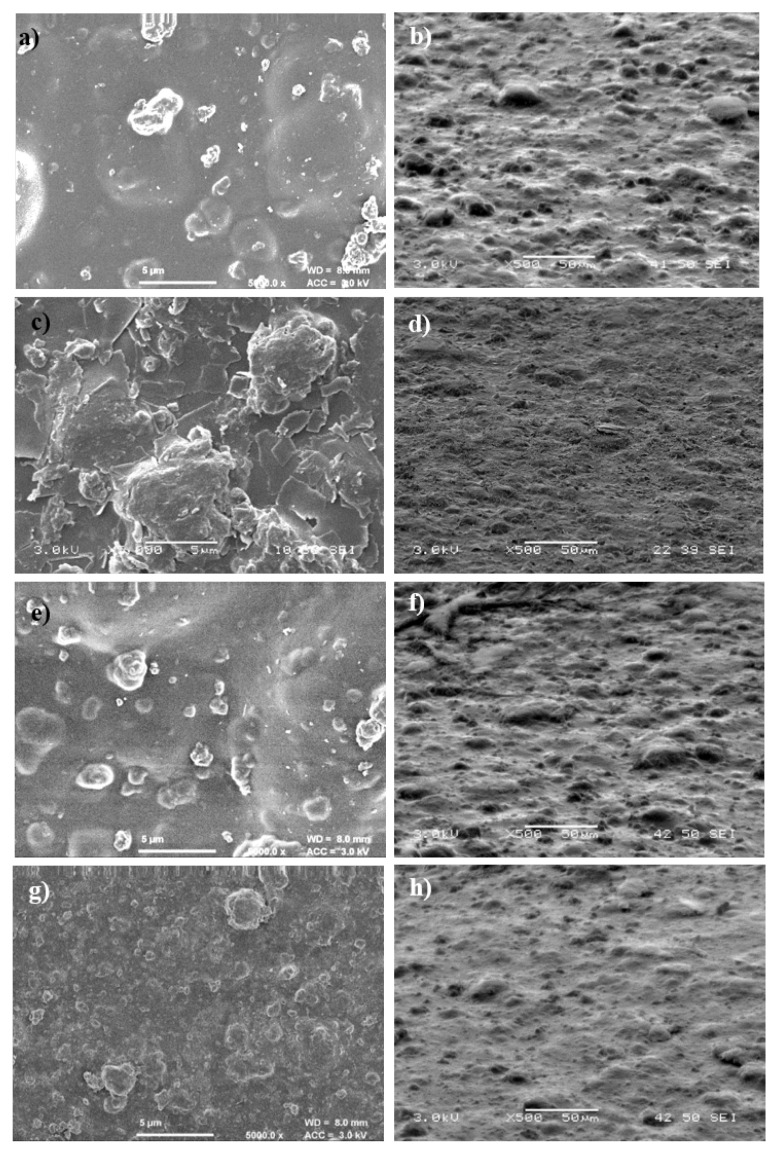
SEM pictures of hydrophobizing agent-coated cellulose–CaSiO_3_ composites (**a**) before coating, after coating with (**c**) TOS, (**e**) E2CA, (**g**) ODPA (scale bar 5 µm) and (**b**,**d**,**f**,**h**) are the corresponding 65° tilted surface images (scale bar 50 µm).

To evaluate the effect of hydrophobizing agents on the surface of the cellulose composites, SEM was used to analyse the surface morphology (Figure 3). The surface morphology is more distinguishable due to the nature of hydrophobic agents used in the hydrophobic process (Figure 3a–h). Figure 3a,b reveal that the uncoated cellulose–CaSiO_3_ composites have a slightly different surface nature than coated samples (Figure 3c–h). After coating, the distribution profile of the TOS treated sample (Figure 3c,d) is much higher than the E2CA- (Figure 3e,f) and ODPA-treated samples (Figure 3g,h). This is could be because the interaction between the cellulose–CaSiO_3_ composite and the hydrophobic agent is more favourable in presence of TOS. This directly correlates with the higher water contact angle values and higher water resistance properties of cellulose–CaSiO_3_ composites coated with TOS.

#### 3.3.5. Thermal Stability Studies of Uncoated and Coated Cellulose–CaSiO_3_ Composites

**Figure 4:**
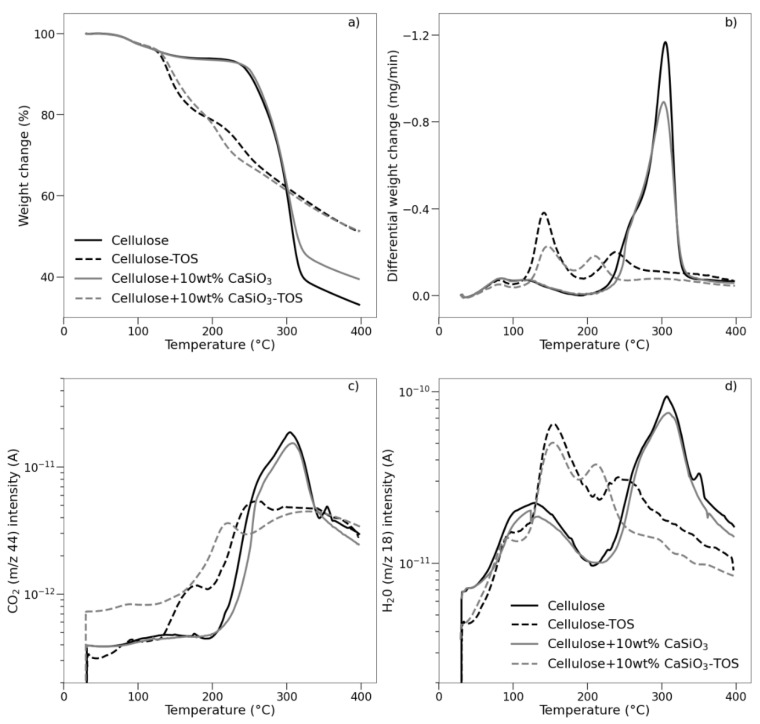
Results of the thermogravimetric analysis of the four cellulose materials (with or without CaSiO_3_, with or without TOS coating): (**a**) weight change versus temperature, (**b**) differential weight change versus temperature, (**c**) mass spectrometer intensity of CO_2_ (*m*/*z* 44) versus temperature, and (**d**) mass spectrometer intensity of H_2_O (*m*/*z* 18) versus temperature.

Thermogravimetry analysis (TGA) under inert gas was used to detect mass changes upon heating; evolved carbon dioxide and water vapour were analysed at the same time using mass spectrometry, because those are the main gases that evolve during the pyrolysis of cellulose. The results are presented in Figure 4. As evidenced by the initial peak around 100 °C in Figure 4d, all samples release water vapour between 80 °C and 120 °C, most likely due to material porosity, resulting in minor weight losses of less than 4 wt%. At higher temperatures, the material stability is different for bare cellulose and TOS-coated cellulose. Cellulose without TOS coating remains fairly stable up to 240 °C when it starts decomposing, with the maximum degradation rate at 305 °C, as can be seen from the differential weight changes (Figure 4b). By 320 °C, the decomposition has slowed down and at 400 °C a char residue of 33.1 wt% is obtained (Figure 4a). This temperature range corresponds to those in the CO_2_ and H_2_O profiles (in Figure 4c,d respectively): both gases start to be detected between 220 °C and 230 °C with a maximum reached at 305 °C. These temperatures are significantly lower than those previously observed with the same type of instrumentation [57,58], but this can be explained by our much slower heating rate of 5 °C/min compared to 50 °C/min in the reported study: any thermal event will then shift towards lower temperatures. Cellulose without TOS coating but containing 10 wt% CaSiO_3_ shows a very similar decomposition profile, with the same temperature range. The only difference is the char residue at 400 °C of 39.5 wt% (Figure 4a). Knowing that there was an amount of 10 wt% of CaSiO_3_ in the starting material, it seems that this compound has mostly not decomposed by 400 °C.

On the other hand, TOS-coated cellulose starts decomposing as early as 120 °C and continuously loses weight until 400 °C. This shows a two-step profile at 140 °C and 237 °C according to the differential weight change in (Figure 4b). By looking at the water profile (Figure 4d), H_2_O is clearly evolved from 120 °C with a first maximum at 150 °C and a second maximum at 230 °C. Accordingly, CO_2_ is released at 130 °C with two maximum temperatures of 175 °C and 240 °C (Figure 4c). Evolving gas profiles are in line with the weight profile, indicating that decomposition occurs significantly sooner in this material than in cellulose with no coating. A char residue of 51% was found at 400 °C (Figure 4a). TOS-coated cellulose containing CaSiO_3_ shows a very similar thermal profile (Figure 4a) with a two-step decomposition starting at 120 °C. The maximum decomposition rates are reached at 146 °C and 211 °C (Figure 4b). Water vapour and carbon dioxide evolve from 120 °C and 150 °C, respectively, and show a two-step profile with comparable peak temperatures to the thermal profile (Figure 4c,d). At the final temperature, a residue of 51.2% was left (Figure 4a).

Generally, the weight loss profile is in line with the evolving gases profile, which leads to the conclusion that the cellulose materials experience pyrolysis even at low temperatures for the TOS-coated samples. The addition of the CaSiO_3_ filler appears to have no effect on cellulose thermal degradation. On the contrary, the surface treatment with TOS shifts the decomposition range towards low temperatures, with a start temperature of 120 °C instead of 240 °C. Chemically TOS is not likely to generate carbon dioxide at these temperatures; however, it could enhance the reduction of the degree of polymerisation, leading to an earlier degradation of cellulose.

#### 3.3.6. Mechanical Property Studies of Uncoated and Coated Cellulose–CaSiO_3_ Composites

**Figure 5:**
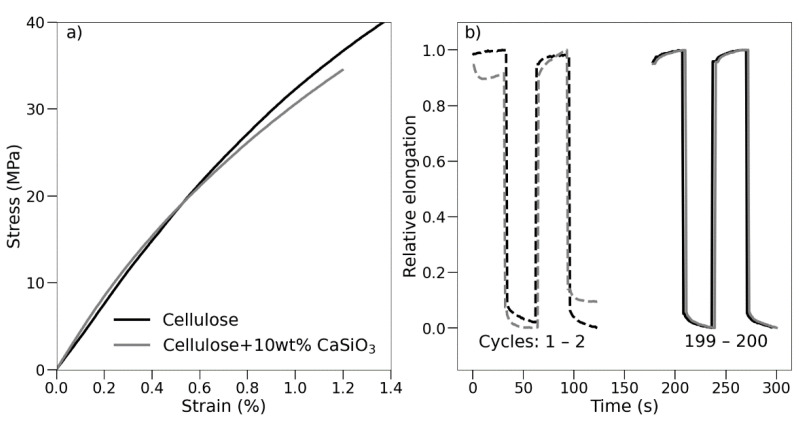
Static mechanical analyses of a cellulose film (**black**) and cellulose–CaSiO_3_ composite (**grey**). (**a**): stress-strain curves at 25 °C. (**b**): Creep cycles showing the relative elongation of the films in the first two cycles on the left (**dashed**) and in the last two ones on the right (**solid**).

A stress–strain diagram is used to describe the deformation of a sample subjected to increasing stress. The stress–strain curves of cellulose with no filler and cellulose with 10 wt% CaSiO_3_ filler are presented in Figure 5a. After a short initial linear range—or Hooke’s region— the stress is no longer proportional to the strain and the slope of the curve decreases. This plastic deformation begins when the strain exceeds 0.15%, which corresponds to a load of 1 N. This low elastic limit is symptomatic of a glassy material film [59,60]. In the initial linear region, the material behaves purely elastically and there is no permanent deformation after removal of the stress. In the following mechanical studies, the sample use was limited to reversible deformation only, with a strain amplitude of 0.1% (DMA) and a stress amplitude of 1 N (creep test). To complete this descriptive study, the creep behaviour of the films was investigated. It consisted of applying a constant load then releasing it, and measuring the induced length variation. This was repeated 200 times, the first and last cycles were compared to determine the possible fatigue of the material. Figure 5b shows the creep curves of a cellulose film and a cellulose film with CaSiO_3_ filler. They both show a purely elastic behaviour and no delay between the application of the load and reaching the maximum elongation. This was the case in the process of releasing the load as well. The materials behaved purely elastically even after 200 cycles with no change in the shape of the creep curves.

**Figure 6:**
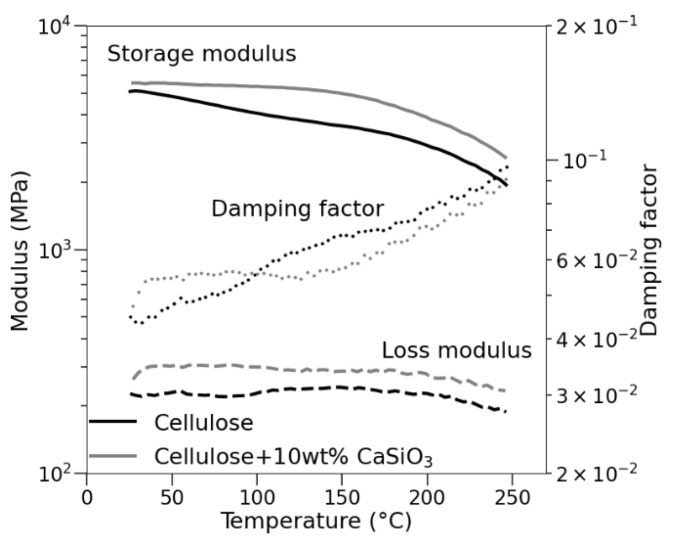
Temperature scan of films made of cellulose only (**black**) and cellulose with 10 wt% CaSiO_3_ (**grey**). The storage modulus (**solid**), the loss modulus (**dashed**) and the damping factor (**dotted**) are plotted versus temperature.

The evolution in temperature of the elastic modulus and its components was then studied. The complex modulus, measured by DMA, represents the degree of stiffness of the material. In Figure 6, the two components of the complex modulus are plotted versus temperature for cellulose and cellulose with 10 wt% CaSiO_3_. A storage modulus of 5 GPa is shown from room temperature to 100 °C, it then decreases slightly to 2 GPa at 250 °C. Regardless of the temperature range, the loss modulus exhibits a lower value of 0.2 GPa. This difference in the order of magnitude is characteristic of glassy materials. From the evolution of the damping factor in Figure 6, it is obvious that no particular transition of the sample material occurs in this temperature range [59,61]. As the elastic modulus is reduced by only a factor of 2 up to 250 °C, it is reasonable to state that the temperature has no detrimental effect on stiffness.

Cellulose films behave as a pure elastic material in the low strain region (below 0.15%) where they have a glassy state from room temperature up to 250 °C. No fatigue behaviour could be observed upon the application of stress. These experiments were useful to qualitatively describe the cellulose films but they are not adapted to a comparison of composition. Instead, the value of the elastic modulus at a given temperature was used as a mechanical descriptor. This was then compared amongst the four cellulose compositions. The elastic modulus of the films was measured by DMA in an environment controlled chamber where the temperature was set to 30 °C while the relative humidity was set at either 20% or 70% RH. These two humidity values correspond to the low-end and high-end values of RH reported in the literature [62]. By following the damping factor, the equilibrium with the surrounding humidity could be detected. It appears that the film needs to absorb moisture at 70% RH for at least 30 min before the damping factor stabilises, but it takes between 30 and 50 min to release humidity to the environment at 20% RH. The elastic modulus was, therefore, measured after 60 min, in a steady damping state.

**Figure 7:**
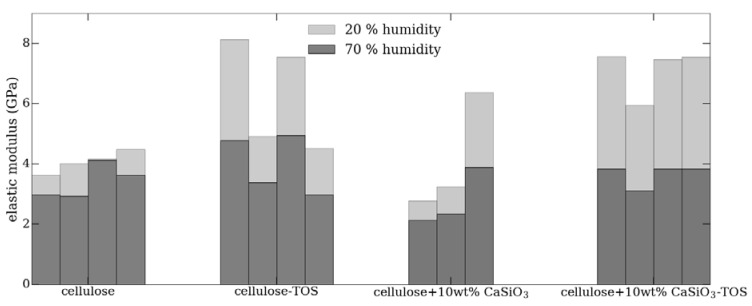
The elastic modulus of cellulose films with various treatments at 20% and 70% relative humidity (RH) (respectively light and dark grey). Each cellulose films were tested on 3 to 4 different films.

Modulus values at 20% and 70% RH are compared in Figure 7 for the various cellulose materials: cellulose with no filler, cellulose with TOS surface treatment, cellulose with 10 wt% CaSiO_3_ composite and coated cellulose-10 wt% CaSiO_3_ composite with TOS surface treatment. It shows that the film stiffness is not affected by the humidity level to the same extent depending on the material. In cellulose with no filler, regardless of the humidity level, there is no significant difference in the values. In contrast, the elastic modulus of the cellulose–CaSiO_3_ composite coated with TOS at 70% RH is half its value at 20% RH, indicating that moisture absorption increases its flexibility. In this material, moisture has a plasticizer effect on cellulose [61]. However, in absolute terms, the elastic modulus of cellulose is still over 2 GPa even at 70% RH. It can be stated that the moisture content in the ambient atmosphere is not detrimental to the mechanical characteristics of the cellulose films. The large sample variability prevents a clear interpretation of the changes in modulus value. Therefore, a statistical analysis of variance (ANOVA) tool was used to determine how much of the measurement variation can be explained by the value of a factor [63]; in this case, the effect of both the presence of a CaSiO_3_ and the surface treatment on the value of the elastic modulus. ANOVA determined that the presence of CaSiO_3_ had no significant effect at the 90% confidence level (*p*-value of 0.36 and 0.28 at 20% and 70% RH, respectively). The surface treatment, however, was proven at 90% confidence to show a significant effect (*p*-value of 0.0036 and 0.085 at 20% and 70% RH, respectively): the effect of surface treatment is an increase in the elastic modulus. Furthermore, no interaction between the two factors was proven at the 90% confidence level.

The mechanical properties of cellulose films have been studied in both static and dynamic modes. Whatever the material composition, the films exhibit glassy behaviour with modulus values ranging from 2 GPa to 6 GPa depending on temperature and humidity, making them suitable for application as a flexible substrate material in electronic devices. Interestingly the mechanical properties are not significantly affected by the presence of CaSiO_3_; however, the surface treatment with TOS appears to increase the modulus value.

### 3.4. Biodegradability Studies of Uncoated and Coated Cellulose–CaSiO_3_ Composites

**Figure 8:**
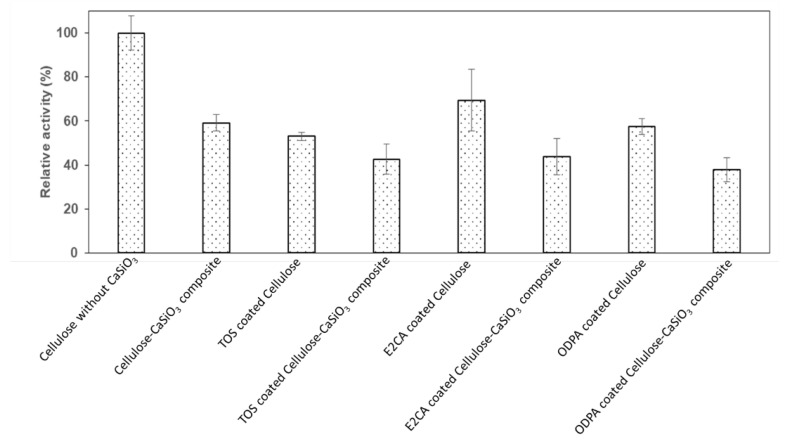
Cellulase activity with different cellulose-based composites as substrates.

The obtained cellulose-based composites were subjected to enzymatic hydrolysis. CaSiO_3_ and the hydrophobizing agents reduced cellulase activity by ~30 to 60% from cellulose without CaSiO_3_ and TOS (Figure 8). Although calcium is not a cellulase inhibitor [64], when CaSiO_3_ was present in the composite, cellulase activity decreased irrespective of the presence or absence of hydrophobic agents. This could be due to the adsorption of cellulases by CaSiO_3_ particles in the composites, which can prevent the interaction between cellulases and cellulose. Furthermore, cellulase activity decreased to 53%, 69% and 57% after the hydrophobic coating performed with TOS, E2CA and ODPA (cellulose without CaSiO_3_), respectively. As previously mentioned, another possible explanation is that the cellulases are also adsorbed into the hydrophobic substrates [65,66,67] and their binding to cellulose is disrupted. This is in good agreement with our recently reported result [54]. Among the various hydrophobic agents used in this work, E2CA has less of an effect on cellulase activities. It can be speculated that this may be due to E2CA forming a hydrophobic porous film on the surface of cellulose [53,68]. However, further research is required to confirm the above biodegradability activities.

## 4. Conclusions

A cellulose-filler composite was prepared using CaSiO_3_ as a filler by a simple dissolution process. EMIMAc was used as an efficient, recoverable and reusable dissolution medium without adding any organic solvent. The addition of CaSiO_3_ into cellulose increases the thermal and flame retardancy properties. However, the prepared material did not meet the required flame retardancy standard and further research is required to improve this behaviour. The hydrophilicity of the cellulose composite was reduced by treating it with efficient hydrophobic agents using a simple dip-coating method. The prepared composite was mechanically stable in the ranges of temperature and humidity from 25 °C to 250 °C and from 20% RH to 70% RH. It showed a purely elastic behaviour with a modulus value between 2 GPa and 6 GPa. Enzyme biodegradation studies on the cellulose composite demonstrated that the hydrophobic agent coating reduced the hydrolysis of cellulose by cellulases. However, cellulases are not completely inactive towards cellulose composites and can be utilised to partially biodegrade the obtained composite. Therefore, the prepared cellulose–CaSiO_3_ composite materials may be suitable as biodegradable substrate materials for the next generation of electronic devices and can pave the way for materials for advanced biodegradable electronic products.

## Figures and Tables

**Table 1:** Effect of the addition of CaSiO_3_ on the LOI and FR properties of cellulose.

Cellulose Composites	LOI (%) (ASTM-D2863)	Flammability Tests UL-94 HB, Time to Burn Marked Area of Samples in Secs (Std. dev) (ASTM D635-03)
No fillers	19	23.7 (±1.2)
10 wt%-CaSiO_3_	20.7	36.3 (±2.8)

## Data Availability

The Data presented in this study are available from the corrosponding authors upon request.

## References

[1] Li G., Zhu R., Yang Y. (2012). Polymer solar cells. Nat. Photonics.

[2] Liu Z., Xu J., Chen D., Shen G. (2015). Flexible electronics based on inorganic nanowires. Chem. Soc. Rev..

[3] Lee J.H., Huynh-Nguyen B.-C., Ko E., Kim J.H., Seong G.H. (2016). Fabrication of flexible, transparent silver nanowire electrodes for amperometric detection of hydrogen peroxide. Sens. Actuators B Chem..

[4] Zeng W., Shu L., Li Q., Chen S., Wang F., Tao X.-M. (2014). Fiber-Based Wearable Electronics: A Review of Materials, Fabrication, Devices, and Applications. Adv. Mater..

[5] Wu X., Ma Y., Zhang G., Chu Y., Du J., Zhang Y., Li Z., Duan Y., Fan Z., Huang J. (2015). Thermally Stable, Biocompatible, and Flexible Organic Field-Effect Transistors and Their Application in Temperature Sensing Arrays for Artificial Skin. Adv. Funct. Mater..

[6] Forrest S.R. (2004). The path to ubiquitous and low-cost organic electronic appliances on plastic. Nature.

[7] Wong W.S., Salleo A. (2009). Electronic Materials: Science & Technology. Flexible Electronics.

[8] Aulin C., Karabulut E., Tran A., Wågberg L., Lindström T. (2013). Transparent Nanocellulosic Multilayer Thin Films on Polylactic Acid with Tunable Gas Barrier Properties. ACS Appl. Mater. Interfaces.

[9] Fujisaki Y., Koga H., Nakajima Y., Nakata M., Tsuji H., Yamamoto T., Kurita T., Nogi M., Shimidzu N. (2014). Transparent Nanopaper-Based Flexible Organic Thin-Film Transistor Array. Adv. Funct. Mater..

[10] Zhang W., Fei L., Zhang J., Chen K., Yin Y., Wang C. (2020). Durable and tunable temperature responsive silk fabricated with reactive thermochromic pigments. Prog. Org. Coat..

[11] Mathur P., Sheikh J.N., Sen K. (2020). Durable flame-retardant wool using sulphamic acid. Polym. Degrad. Stab..

[12] Shokri J., Adibki K. (2013). Application of Cellulose and Cellulose Derivatives in Pharmaceutical Industries. Cellulose—Medical, Pharmaceutical and Electronic Applications.

[13] Song Z., Xiao H., Zhao Y. (2014). Hydrophobic-modified nano-cellulose fiber/PLA biodegradable composites for lowering water vapor transmission rate (WVTR) of paper. Carbohydr. Polym..

[14] Jabbour L., Bongiovanni R., Chaussy D., Gerbaldi C., Beneventi D. (2013). Cellulose-based Li-ion batteries: A review. Cellulose.

[15] Danafar F., Kalantari M. (2018). A Review of Natural Rubber Nanocomposites Based on Carbon Nanotubes. J. Rubber Res..

[16] Huang W., Ling S., Li C., Omenetto F.G., Kaplan D.L. (2018). Silkworm silk-based materials and devices generated using bio-nanotechnology. Chem. Soc. Rev..

[17] Dutta S., Kim J., Ide Y., Ho Kim J., Hossain M.S.A., Bando Y., Yamauchi Y., Wu K.C.W. (2017). 3D network of cellulose-based energy storage devices and related emerging applications. Mater. Horiz..

[18] Zhao D., Zhu Y., Cheng W., Chen W., Wu Y., Yu H. (2020). Cellulose-Based Flexible Functional Materials for Emerging Intelligent Electronics. Adv. Mater..

[19] Gao L., Chao L., Hou M., Liang J., Chen Y., Yu H.-D., Huang W. (2019). Flexible, transparent nanocellulose paper-based perovskite solar cells. NPJ Flex. Electron..

[20] Zhu H., Xiao Z., Liu D., Li Y., Weadock N.J., Fang Z., Huang J., Hu L. (2013). Biodegradable transparent substrates for flexible organic-light-emitting diodes. Energy Environ. Sci..

[21] Khan A., Khan F.R., Kim H.S. (2018). Electro-Active Paper as a Flexible Mechanical Sensor, Actuator and Energy Harvesting Transducer: A Review. Sensors.

[22] Vicente A.T., Araújo A., Mendes M.J., Nunes D., Oliveira M.J., Sanchez-Sobrado O., Ferreira M.P., Águas H., Fortunato E., Martins R. (2018). Multifunctional cellulose-paper for light harvesting and smart sensing applications. J. Mater. Chem. C.

[23] Jose J., Thomas V., Vinod V., Abraham R., Abraham S. (2019). Nanocellulose based functional materials for supercapacitor applications. J. Sci. Adv. Mater. Devices.

[24] Sirviö J.A., Heiskanen J.P. (2020). Room-temperature dissolution and chemical modification of cellulose in aqueous tetraethylammonium hydroxide–carbamide solutions. Cellulose.

[25] Xu A., Cao L., Wang B., Ma J. (2015). Dissolution Behavior of Cellulose in IL + DMSO Solvent: Effect of Alkyl Length in Imidazolium Cation on Cellulose Dissolution. Adv. Mater. Sci. Eng..

[26] Isogai A., Atalla R.H. (1995). Alkaline Method for Dissolving Cellulose. U.S. Patent.

[27] Olsson C., Westman G. (2013). Direct Dissolution of Cellulose: Background, Means and Applications. Cellulose—Fundamental Aspects.

[28] Zhang S., Li F.-X., Yu J., Hsieh Y.-L. (2010). Dissolution behaviour and solubility of cellulose in NaOH complex solution. Carbohydr. Polym..

[29] Rodríguez-Castellanos W., Martínez-Bustos F., Rodrigue D., Trujillo-Barragán M. (2015). Extrusion blow molding of a starch–gelatin polymer matrix reinforced with cellulose. Eur. Polym. J..

[30] Faruk O., Bledzki A.K., Fink H.-P., Sain M. (2012). Biocomposites reinforced with natural fibers: 2000–2010. Prog. Polym. Sci..

[31] Zhu S., Wu Y., Chen Q., Yu Z., Wang C., Jin S., Ding Y., Wu G. (2006). Dissolution of cellulose with ionic liquids and its application: A mini-review. Green Chem..

[32] Swatloski R.P., Spear S.K., Holbrey J.D., Rogers R.D. (2002). Dissolution of Cellose with Ionic Liquids. J. Am. Chem. Soc..

[33] Zavrel M., Bross D., Funke M., Büchs J., Spiess A.C. (2009). High-throughput screening for ionic liquids dissolving (ligno-)cellulose. Bioresour. Technol..

[34] Payal R.S., Balasubramanian S. (2014). Dissolution of cellulose in ionic liquids: An ab initio molecular dynamics simulation study. Phys. Chem. Chem. Phys..

[35] Sun N., Rahman M., Qin Y., Maxim M.L., Rodríguez H., Rogers R.D. (2009). Complete dissolution and partial delignification of wood in the ionic liquid 1-ethyl-3-methylimidazolium acetate. Green Chem..

[36] Liebert T., Heinze T. (2008). Interaction of ionic liquids wlth polysaccharides 5. Solvents and reaction media for the modification of cellulose. BioResources.

[37] Yang Z., Zhang Y., Fu F., Liu X. (2017). Single-faced flame resistance of cotton fabrics modified via mist copolymerization. RSC Adv..

[38] Mohamed A.L., Hassabo A.G. (2015). Flame Retardant of Cellulosic Materials and Their Composites. Flame Retardants Electronic.

[39] Nakanishi S., Masuko F., Hori K., Hashimoto T. (2000). Pyrolytic Gas Generation of Cotton Cellulose With and Without Flame Retardants at Different Stages of Thermal Degradation: Effects of Nitrogen, Phosphorus, and Halogens. Text. Res. J..

[40] Salmeia K., Gaan S., Malucelli G. (2016). Recent Advances for Flame Retardancy of Textiles Based on Phosphorus Chemistry. Polymers.

[41] Alongi J., Malucelli G. (2015). Cotton flame retardancy: State of the art and future perspectives. RSC Adv..

[42] Gao W.-W., Zhang G.-X., Zhang F.-X. (2015). Enhancement of flame retardancy of cotton fabrics by grafting a novel organic phosphorous-based flame retardant. Cellulose.

[43] Zheng D., Zhou J., Wang Y., Zhang F., Zhang G. (2018). A reactive flame retardant ammonium salt of diethylenetriaminepenta(methylene-phosphonic acid) for enhancing flame retardancy of cotton fabrics. Cellulose.

[44] Zheng C., Li D., Ek M. (2019). Improving fire retardancy of cellulosic thermal insulating materials by coating with bio-based fire retardants. Nord. Pulp Pap. Res. J..

[45] Teisala H., Tuominen M., Kuusipalo J. (2014). Superhydrophobic Coatings on Cellulose-Based Materials: Fabrication, Properties, and Applications. Adv. Mater. Interfaces.

[46] Bretel G., Rull-Barrull J., Nongbe M.C., Terrier J.-P., Le Grognec E., Felpin F.-X. (2018). Hydrophobic Covalent Patterns on Cellulose Paper through Photothiol-X Ligations. ACS Omega.

[47] Nyström D., Lindqvist J., Östmark E., Hult A., Malmström E. (2006). Superhydrophobic bio-fibre surfaces via tailored grafting architecture. Chem. Commun..

[48] Wang T., Hu X., Dong S. (2007). A general route to transform normal hydrophilic cloths into superhydrophobic surfaces. Chem. Commun..

[49] Ghose T.K. (1987). Measurement of cellulase activities. Pure Appl. Chem..

[50] Xu J., Zhang L., Chen G. (2013). Fabrication of graphene/poly(ethyl 2-cyanoacrylate) composite electrode for amperometric detection in capillary electrophoresis. Sens. Actuators B Chem..

[51] Han M.G., Kim S., Liu S.X. (2008). Synthesis and degradation behavior of poly(ethyl cyanoacrylate). Polym. Degrad. Stab..

[52] Bayer I.S., Fragouli D., Attanasio A., Sorce B., Bertoni G., Brescia R., Di Corato R., Pellegrino T., Kalyva M., Sabella S. (2011). Water-Repellent Cellulose Fiber Networks with Multifunctional Properties. ACS Appl. Mater. Interfaces.

[53] Du X., Li J.S., Li L.X., Levkin P.A. (2013). Porous poly(2-octyl cyanoacrylate): A facile one-step preparation of superhydrophobic coatings on different substrates. J. Mater. Chem. A.

[54] Chandrasekaran S., Sotenko M., Cruz-Izquierdo A., Rymansaib Z., Iravani P., Kirwan K., Scott J.L. (2021). Preparation of Printable and Biodegradable Cellulose-Laponite Composite for Electronic Device Application. J. Polym. Environ..

[55] Marcinko S., Fadeev A.Y. (2004). Hydrolytic Stability of Organic Monolayers Supported on TiO_2_ and ZrO_2_. Langmuir.

[56] Sha Y., Deng C., Liu B. (2008). Development of C18-functionalized magnetic silica nanoparticles as sample preparation technique for the determination of ergosterol in cigarettes by microwave-assisted derivatization and gas chromatography/mass spectrometry. J. Chromatogr. A.

[57] Grønli M., Antal M.J., Várhegyi G. (1999). A Round-Robin Study of Cellulose Pyrolysis Kinetics by Thermogravimetry. Ind. Eng. Chem. Res..

[58] Statheropoulos M., Kyriakou S.A. (2000). Quantitative thermogravimetric-mass spectrometric analysis for monitoring the effects of fire retardants on cellulose pyrolysis. Anal. Chim. Acta.

[59] Ruan D., Zhang L., Zhang Z., Xia X. (2004). Structure and properties of regenerated cellulose/tourmaline nanocrystal composite films. J. Polym. Sci. Part B Polym. Phys..

[60] Hsieh Y.-C., Yano H., Nogi M., Eichhorn S.J. (2008). An estimation of the Young’s modulus of bacterial cellulose filaments. Cellulose.

[61] Zhou S., Tashiro K., Hongo T., Shirataki H., Yamane C., Ii T. (2001). Influence of Water on Structure and Mechanical Properties of Regenerated Cellulose Studied by an Organized Combination of Infrared Spectra, X-ray Diffraction, and Dynamic Viscoelastic Data Measured as Functions of Temperature and Humidity. Macromolecules.

[62] Peixoto J., Oort A.H. (1996). The Climatology of Relative Humidity in the Atmosphere. J. Clim..

[63] Brynn Hibbert D., Gooding J.J. (2006). Data analysis for chemistry: An introductory guide for students and laboratory scientists. Choice Rev. Online.

[64] Tejirian A., Xu F. (2010). Inhibition of Cellulase-Catalyzed Lignocellulosic Hydrolysis by Iron and Oxidative Metal Ions and Complexes. Appl. Environ. Microbiol..

[65] Sammond D.W., Yarbrough J.M., Mansfield E., Bomble Y.J., Hobdey S.E., Decker S.R., Taylor L.E., Resch M.G., Bozell J.J., Himmel M.E. (2014). Predicting Enzyme Adsorption to Lignin Films by Calculating Enzyme Surface Hydrophobicity. J. Biol. Chem..

[66] Tu M., Pan X., Saddler J.N. (2009). Adsorption of Cellulase on Cellulolytic Enzyme Lignin from Lodgepole Pine. J. Agric. Food Chem..

[67] Lu Y., Yang B., Gregg D., Saddler J.N., Mansfield S.D. (2002). Cellulase Adsorption and an Evaluation of Enzyme Recycle During Hydrolysis of Steam-Exploded Softwood Residues. Appl. Biochem. Biotechnol..

[68] Korde J.M., Kandasubramanian B. (2018). Biocompatible alkyl cyanoacrylates and their derivatives as bio-adhesives. Biomater. Sci..

